# Consumer acceptance of new food trends resulting from the fourth industrial revolution technologies: A narrative review of literature and future perspectives

**DOI:** 10.3389/fnut.2022.972154

**Published:** 2022-08-10

**Authors:** Abdo Hassoun, Janna Cropotova, Monica Trif, Alexandru Vasile Rusu, Otilia Bobiş, Gulzar Ahmad Nayik, Yash D. Jagdale, Farhan Saeed, Muhammad Afzaal, Parisa Mostashari, Amin Mousavi Khaneghah, Joe M. Regenstein

**Affiliations:** ^1^Sustainable AgriFoodtech Innovation and Research (SAFIR), Arras, France; ^2^Syrian Academic Expertise (SAE), Gaziantep, Turkey; ^3^Department of Biological Sciences Ålesund, Norwegian University of Science and Technology, Ålesund, Norway; ^4^Department of Food Research, Centre for Innovative Process Engineering (CENTIV) GmbH, Syke, Germany; ^5^Life Science Institute, University of Agricultural Sciences and Veterinary Medicine Cluj-Napoca, Cluj-Napoca, Romania; ^6^Genetics and Genetic Engineering, Faculty of Animal Science and Biotechnology, University of Animal Sciences and Veterinary Medicine Cluj-Napoca, Cluj-Napoca, Romania; ^7^Animal Science and Biotechnology Faculty, University of Agricultural Sciences and Veterinary Medicine Cluj-Napoca, Cluj-Napoca, Romania; ^8^Department of Food Science and Technology, Government Degree College, Shopian, India; ^9^MIT School of Food Technology, MIT ADT University, Pune, India; ^10^Department of Food Sciences, Government College University Faisalabad, Faisalabad, Pakistan; ^11^Department of Food Science and Technology, Faculty of Nutrition Sciences and Food Technology, National Nutrition and Food Technology Research Institute, Shahid Beheshti University of Medical Sciences, Tehran, Iran; ^12^Department of Fruit and Vegetable Product Technology, Prof. Wacław Dabrowski Institute of Agricultural and Food Biotechnology – State Research Institute, Warsaw, Poland; ^13^Department of Food Science, Cornell University, Ithaca, NY, United States

**Keywords:** alternative proteins, edible insects, cultured meat, consumer perception, plant-based food, 3D food printing, personalized nutrition, industry 4.0

## Abstract

The growing consumer awareness of climate change and the resulting food sustainability issues have led to an increasing adoption of several emerging food trends. Some of these trends have been strengthened by the emergence of the fourth industrial revolution (or Industry 4.0), and its innovations and technologies that have fundamentally reshaped and transformed current strategies and prospects for food production and consumption patterns. In this review a general overview of the industrial revolutions through a food perspective will be provided. Then, the current knowledge base regarding consumer acceptance of eight traditional animal-proteins alternatives (e.g., plant-based foods and insects) and more recent trends (e.g., cell-cultured meat and 3D-printed foods) will be updated. A special focus will be given to the impact of digital technologies and other food Industry 4.0 innovations on the shift toward greener, healthier, and more sustainable diets. Emerging food trends have promising potential to promote nutritious and sustainable alternatives to animal-based products. This literature narrative review showed that plant-based foods are the largest portion of alternative proteins but intensive research is being done with other sources (notably the insects and cell-cultured animal products). Recent technological advances are likely to have significant roles in enhancing sensory and nutritional properties, improving consumer perception of these emerging foods. Thus, consumer acceptance and consumption of new foods are predicted to continue growing, although more effort should be made to make these food products more convenient, nutritious, and affordable, and to market them to consumers positively emphasizing their safety and benefits.

## Introduction

The global challenges for economic, social, and environmental sustainable development are currently more acute than ever before and call for immediate actions to develop a healthier and more sustainable future of food (1–3). Food production systems, mainly the production of animal-sourced food through livestock farming, have been a significant contributor to climate change and unsustainable development. Therefore, a search is underway worldwide to find alternative technologies and production methods that provide food with a lower environmental footprint while nutritional and sensory characteristics are similar or even better than that of animal products (4–8).

Plant-based sources have been investigated and established for use as food and feed throughout human development, but consumer interest in plant-based proteins has recently increased, which is reflected in a growing number of vegans, vegetarians, or flexitarians. A variety of plant-based meat, fish, milk, and egg analogs are being introduced to the market as a promising, sustainable approach to reduce the consumption of meat and other animal-based proteins (9, 10). While wild-harvested insects have been a traditional food source in many countries for centuries (11), insects' cultivation is relatively new, with some small-scale insect farming projects being launched in some countries (12). Apart from these traditional sources (i.e., plant-based foods and insects), other more innovative solutions, especially cell-cultured and 3D printed-foods, are being evaluated. Cell-cultured food production (e.g., meat, seafood, and poultry) is being studied owing to its potential to achieve environmental sustainability, due to low land and water requirements and reduced greenhouse gas emissions as well as improved animal welfare (13–15). 3D printing is a new technique that has become part of many scientific fields and industrial areas, including the food industry, allowing the production of on-demand, complex, and customized foods. In addition, the technique may be used for personalized diet (or personalized foods) to print products that specifically meet an individual's health-nutritional needs (16, 17). Another emerging application of 3D printing is cultured meat (18).

Innovative technologies have the potential to improve food production and enhance the quality of new food products to improve consumer acceptance. Gene editing is one of the emerging technologies that have opened up many possibilities for generating crops and animals with improved properties and desired traits (19–21). Additionally, highly productive food production systems (e.g., hydroponics, aquaponics, and aeroponics) have received attention as alternative farming systems, taking advantages of innovations and advancements in science and technology (22–24). Increased concerns about environmental sustainability are driving the growing interest in better uses of food wastes, by-products, and ugly produce. Food wastes is one of the major challenges for the global food system as approximately one-third of food produced in the world for human consumption is either lost or wasted each year. Valorization of food by-products and ugly produce (e.g., food products with an abnormal appearance) using smart solutions and technologies can constitute a promising strategy to tackle this challenge (25–27).

**Figure 1:**
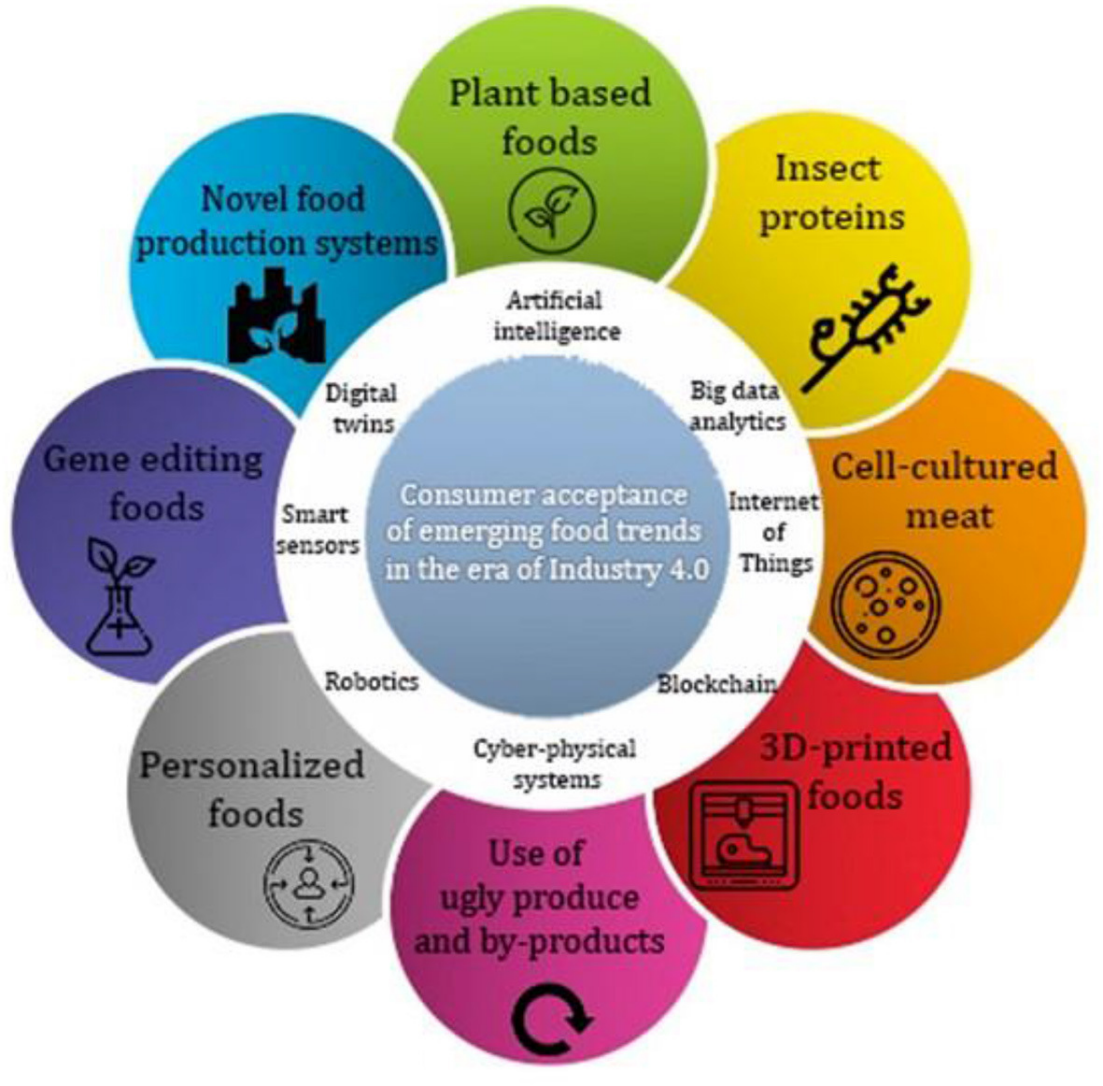
The food trends reviewed in this manuscript and the main enablers of the fourth industrial revolution.

**Figure 2:**
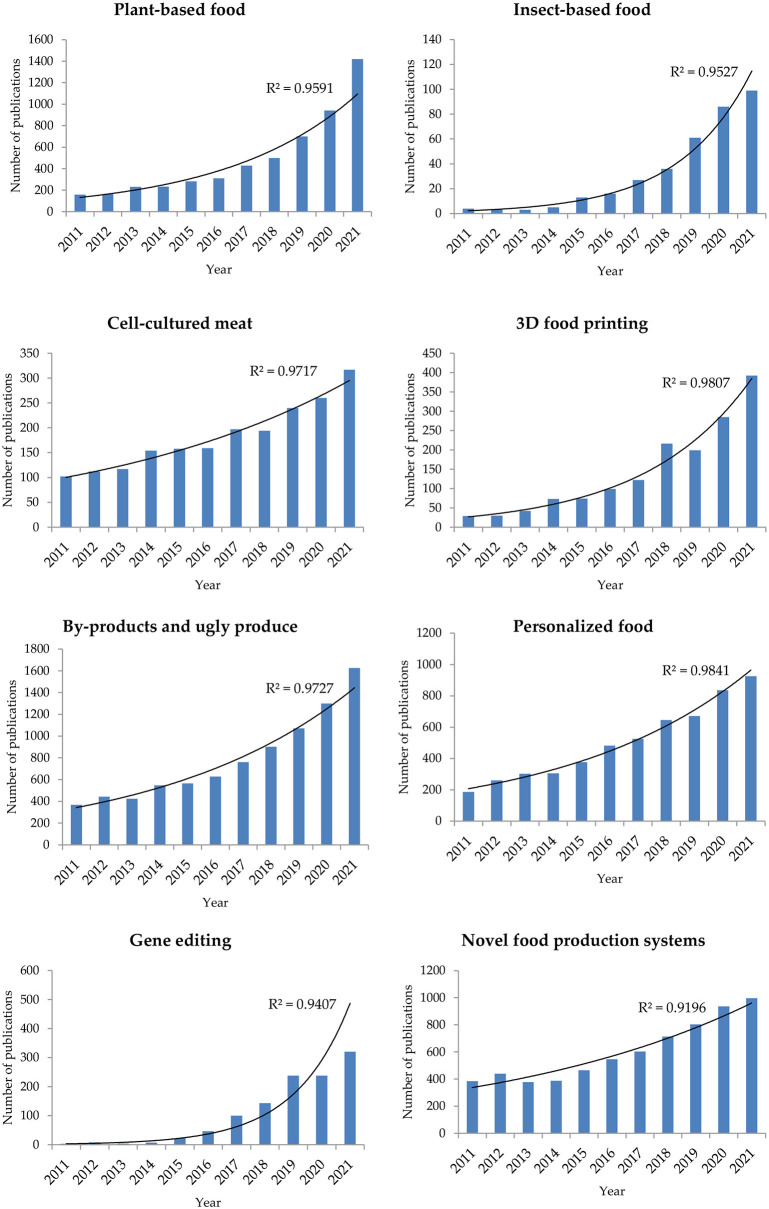
Number of publications per year (until March 2022) dealing with emerging food trends (data obtained from Scopus).

The evolution of consumers' demands for the aforementioned eight food trends, namely, plant-based, insect-based, cell-based, 3D-printed, personalized, and gene-edited foods, as well as foods resulting from by-products and ugly produce and new production systems (Figure 1) has resulted in a complexity that requires advanced technologies and innovative solutions. There is a growing literature on these selected food trends as can be seen in Figure 2. These trends have been further fueled by recent technological innovations accompanied by the advent of the fourth industrial revolution (or Industry 4.0) technologies. Due to its complexity, it is difficult to provide a single, concise definition of Industry 4.0 that will be universally accepted. However, Industry 4.0 can be seen as a combination of smart and advanced technologies in the digital, physical, and biological fields that enables more advanced intelligence to be brought to manufacturing and the transition from mass to customized production (28, 29). The main Industry 4.0 enablers in the food industry include artificial intelligence (AI), big data, the Internet of Things (IoT), blockchain, smart sensors, robotics, digital twins, and cyber-physical systems (30, 31).

Plant-based foods have been thoroughly reviewed in recent publications (10, 32–35). Detailed review papers reporting on insects protein (11, 36–38), cell-cultured (13–15), and 3D printed (39–41) food products have also been published. Other publications reported on personalized diet (16, 17, 42, 43), gene editing technologies (19, 20, 44), valorization of food by-products and ugly produce (45–48), and new food production systems (22, 24, 49). However, the applicability of Industry 4.0 concepts with each of these food trends has not been reviewed. Therefore, the main objective of this narrative review is to highlight the important scientific and technological advances that are being used to improve sensory, nutritional, and technological qualities of emerging food trends (shown in Figure 1), and enhancing their acceptance by consumers.

## Overview of the industrial revolutions through a food perspective

The ever-increasing population is putting pressure on natural resources and depleting them rapidly. Technological, environmental, social, and political changes across the globe are creating many new opportunities and challenges for humans. The global population increase is significantly affecting food and water sources (50). The overall industrial revolutions have had a great impact on the many different components of the food industry. The food industry has been continuously updating its processes and products to meet each new revolution (51). Industry 4.0 comprises a diversity of new enabling technologies, as previously mentioned that includes smart sensors, big data, AI, IoT, blockchain, cloud computing, automation, among others. These technologies have important roles in creating modern production processes. The food industry is adopting a customer-orientation as part of a dynamic supply chain. An adaptation of innovative technologies in the different food sectors is important for the sustainability of the production process. New technologies are often also more efficient economically (52, 53).

The United Nations is striving to make the environment and food production more sustainable for upcoming generations. The industrial revolutions are important factors for sustainable food production and the environment (54–56). Industry 4.0 has a direct impact on food manufacturing and the food supply chain. Industry 4.0 is integrating human actors and intelligent machines with product and process lines. Consumer demand for healthy food will best be provided by adopting Industry 4.0 changes. For example, thermal food processing is being replaced with non-thermal technologies to minimize nutrient losses (56). The use of non-thermal technologies (e.g., high-pressure processing (HPP), cold plasma (CPL), and pulsed UV-light) are improving processing to produce safe and more nutritious food products (57).

Automation is having an increasingly important role in manufacturing to achieve maximum productivity. The use of AI with automated processes is increasing in the food industry (54). AI helps monitor the supply chain and overall production process. IoT includes many technologies that will also affect existing production processes. IoT could also be implemented in the food supply chain to make food safer (58). IoT connects different devices to ensure effective communications between people and things (59). The use of sensor technology and cloud computing devices are important in increasing the efficiency of the food supply chain (60, 61). The application of Industry 4.0 technologies (e.g., IoT, blockchain, and smart sensors) is also important in reducing food wastage (62). Generally, the digital revolution currently occurring in manufacturing and the food industry, accompanied by greater automation and advanced monitoring methods and processing technologies is likely to have significant roles in enhancing sensory quality and nutritional properties of foods, leading to improved consumer perception and acceptance of these foods.

## Consumer acceptance of emerging food trends

### Plant-based foods

Current food production practices have been linked to a high prevalence of various chronic diseases as well as significant environmental damage (63, 64). Over the past century, the modern food and agricultural sectors have contributed to a considerable reduction in world malnutrition and hunger by producing a bountiful supply of inexpensive, safe, and tasty foods. To feed a rising and wealthier global population, more food of higher quality is required. Large-scale production of animal products such as milk, fish, meat, eggs, and their derivatives have been identified as a major contributor to the modern food supply's negative impact on global environmental sustainability (63). Raising cattle for food causes significantly more pollution, water and land use, greenhouse gas emissions, and biodiversity loss than growing plants (and in some cases other animals) for human use (65).

Plant-based (PB) diets are becoming increasingly popular as a strategy to lessen the diet's environmental footprint while simultaneously improving human health and animal welfare. In comparison to omnivores, vegetarians and vegans make up a small percentage of the population, but their numbers have risen in recent years. A side from meat alternatives, non-animal food products are becoming more popular, which creates a business opportunity for the food industry (66). Concerns over the consumption of animal-based food products and their harmful effects on the environment and health have led to an increase in the PB protein business, particularly for innovative items that can replace traditional dairy, egg, and meat products. More people are declaring themselves “flexitarians (vegetarians who occasionally eat animal products),” or opting to consume less dairy, eggs, and meat in favor of more PB meals to help the environment, improve health, or both. According to consumer market research, up to 5 million Americans will have given up meat totally between 2019 and 2020, becoming vegetarians or vegans (67) although data confirming this is not yet available.

Functional PB foods are produced from unprocessed or natural, as well as biotechnologically modified plants. They are considered to have a significant impact on health and wellbeing by reducing disease risks. Many of these functional foods have been related to lower incidences of a variety of health conditions, including diabetes, cardiovascular disease, gout, and cancer. As a result, there is rising interest in functional PB food research and development (68, 69). Individual PB foods, such as nuts, vegetables, fruits, legumes, whole grains, and coffee, have been shown to be beneficial to the cardiovascular system (70). Significant evidence, on the other hand, links particular animal foods, such as processed and red meat, to an elevated risk of cardiovascular diseases (71, 72), although these results remain controversial. Consumers are increasingly turning to PB milk replacements for health reasons such as lactose intolerance, cow's milk protein allergies, or as a lifestyle choice. PB milk substitutes are generally water-soluble extracts of oilseeds, legumes, pseudo-cereals, or cereals that resemble bovine milk in appearance. As a substitute for cow's milk, they are manufactured by reducing the raw material's size, extracting it in water, and then homogenizing it. Cow's milk replacers can be used as a straight replacement for cow's milk or in some animal milk-based recipes (73) although their nutritional profiles may be quite different and this remains a concern.

Furthermore, there is growing concern that animal waste lagoons and industrial meat production runoff would pollute natural resources such as rivers, streams, and drinking water although manure also represents a potential natural fertilizer. There is also concern that excessive livestock farming may result in the loss of critical carbon sinks such as forests and other regions, as well as increased greenhouse gas emissions, which will exacerbate current environmental and climate-related issues. For human health and natural resources reasons a sustainable food system that shifts the world population toward less animal-based foods and more PB foods is potentially beneficial. Dietary patterns rich in minimally processed whole grains, vegetables, fruits, nuts, and legumes have been recommend for increased sustainability and human health. Meanwhile, a variety of other PB food products have been developed to replace traditional animal-based foods such as meat alternatives, e.g., sausages, burgers, and other meat-like products made primarily from highly processed PB components. Even though these products provide more PB alternatives, they may or may not be intended to imitate the sensory experience of eating meat (65, 71, 72).

The number of people consuming PB diets is rapidly expanding, according to many vegan organizations and consulting firms, although some recent reports suggest that the rate of growth of this market segment may be slowing down as repeat purchases decrease. It is claimed that vegans in the United States increased by 500% from almost 4 million in 2014 to 19.6 million in 2017 (66). According to a national survey done in the United States in 2018, Americans had been reducing their meat consumption in the previous 3 years (74). In the United Kingdom, flexitarians account for 21% of the population, whereas vegetarians and vegans account for one in every eight people. In Germany, vegetarians went from 1% (2005) to 7% (2018); the meat-free population expanded by 94.4% from 2011 to 2016 in Italy, and flexitarians increased by 25% in 2 years in Spain (66). Furthermore, according to global research done in 2019, 40% of consumers are attempting to reduce animal protein consumption, with 10% having completely stopped eating red meat (75). PB meat substitutes are predicted to expand in value from USD 1.6 billion in 2019 to USD 3.5 billion by 2026. The top-selling meat replacement foods in 2019 were burgers (USD 283 million), hot dogs and sausages (USD 159 million), and patties (USD 120 million). Other figures show that sales of meat in the United States fell by 5% between 2015 and 2019 (66, 75).

Due to an increase in information about chronic diseases and the numerous health claims presently associated with various foods, consumer interest in wellness and better health is expanding. Nowadays, many consumers drink PB milk substitutes because they want to rather than because they have an allergy (76). PB milk substitutes are often regarded as healthy, owing to their established health claims, such as those relating to vitamins, fiber, or no cholesterol. The market is being driven by both these positive attributes as well as people's negative perceptions about cow's milk. The possibility of cow's milk contributing to a variety of human ailments, as well as its high-fat content, are among the concerns (76). The market for PB milk replacements has also grown significantly, more than tripling its global sales from 2009 to 2015 and reaching 21 billion USD (73). According to the Plant Based Foods Association, sales of PB yogurts have increased by 55%, PB cheeses by 43%, and PB creamers by 131% in the United States (66).

The Industry 4.0 food processing technologies improve functional, nutritional, and sensory properties of new PB foods. Non-thermal technologies such as PEF, HPP, high-pressure homogenization, and ultrasound modify the permeability of the cell membrane in numerous fruits and vegetables. This has been connected to microstructural changes in the whole matrices and reduced particle size in liquid matrices. In general, this increases the bioavailability of phenolic and carotenoids compounds by promoting their release (77). Furthermore, this type of processing might be effective in addressing the obstacles that come with processing PB drinks on a larger scale (69). An innovative drying processing technique–intermittent drying, is a method of changing the drying conditions by varying the humidity, temperature, pressure, velocity, or even the heat input mode. Longer drying durations and case hardening decrease energy efficiency, and lead to poor quality attributes that have been successfully addressed with this drying procedure in different PB foods (78).

The 3D-printing of PB foods has the potential to produce better quality PB foods. The purpose of 3D printing is to turn a computer-aided design model into a three-dimensional object. 4D printing is a relatively new technology that complements 3D printing by allowing the printed material to alter over time. Food 3D printing has the unique ability to create geometrically complex structures that can be mass produced while also saving money and the environment. It allows for the customization of foods based on nutritional needs, calorie consumption, texture, a precise shape, flavor, or color. For example, extrusion, selective sintering, binder jetting, and inkjets are the four types of 3D food printing technologies currently being studied for PB foods (79).

### Insect-based foods

In response to the increase in the world's population, the existing production of food will have to treble to fulfill the rapidly rising demand for food. Insects are being researched as a new source of animal feed and human food to help meet global food security challenges. Human consumption of insects has several reported advantages including comparable protein levels (80), relatively high levels of unsaturated fat and different nutrients, and a lesser environmental effect due to decreased greenhouse gas emissions (80, 81). Insects are regarded as more sustainable since they utilize fewer natural resources such as water, feed, and land, and they generate far fewer greenhouse gases and ammonia than bovine and non-bovine animals. They have a high feed conversion ratio because they are cold-blooded, implying that they are particularly efficient at bio-transforming organic resources into insect biomass (82, 83).

As a result, insect production for human food is increasing in several countries (84). Around 2,000 edible insect species have been identified worldwide. They have been collected from the wild including from Africa, East Asia, and South America, and are used in traditional diets (37). Beetles (31%), caterpillars (18%), ants, wasps, and bees (14%), cricket, locusts, and grasshoppers (Orthoptera) (13%), planthoppers, cicadas, scale insects, true bugs (Hemiptera), and leafhoppers (10%), termites (Isoptera) (3%), dragonflies (Odonata) (3%); flies (Diptera) (2%); and other orders (5%) are the most commonly consumed species globally (85). For example cricket powder was added to pasta to increase its content of protein and minerals and improved the culinary properties and texture (86).

Insect-based food production has been influenced by recent advances and innovations offered by Industry 4.0 technologies. One of them is exploiting the possibilities for engineered insect tissue in cellular culture. Cellular agriculture is a rapidly emerging field that allows for the preparation of such a food system without necessitating changes in customer behavior. The use of insect cell culture in cellular agriculture offers the promise to overcome technical limitations and produce low-input, high-volume, and nutritious food. Insect cells are good candidates for incorporation into cultured meat and other innovative food products due to the robustness of established techniques for culturing insect cells and their ease of immortalization, serum-free growth, have a high-density proliferation, transfection, and a good suspension culture adaptation compared to mammalian cells (87).

Despite their apparent feasibility as a long-term alternative to conventional protein sources, there are still several barriers to their widespread utilization as human food in the West (81). In many Western countries, consumer acceptance remains a hurdle, and insects are usually viewed as unpleasant, even though their flavor has been shown to be mild and tolerable. Consumer disgust can be explained in numerous ways, including social, cultural and religious reasons (84). Therefore, product development to create new insect-based foods, as well as acceptance-boosting strategies, are required (88).

Consumer acceptance of insect-based food has been studied (89–91). For example, food neophobia, or a fear of trying new foods, emerges as an evolutionary response to prevent potential hazards from being tried. Many aspects of human eating behavior, including dietary preferences and food choices, are influenced by this attitude. Consumers in countries where there has been no recent insect intake history have a particularly neophobic attitude toward edible insects, which influences their apprehension to consume unusual and perhaps repulsive foods like insects (89). For example, the Chinese had more favorable attitudes and reported a higher willingness to eat insects compared to the Germans (90). Sensory aversion was discovered to be one of the commonly recognized risks of insect intake, which affects both Indians and Americans (89). Consumer acceptance of insect-based foods remains a hurdle in many cultures, where religious prohibitions rule out many insects. The role of context (social companions and location) in the acceptance of insect-based foods was studied (91). The results showed that eating with friends and eating in pubs enhanced the acceptance of insect-based foods. In another study (92), names and visual presentations were found to be important factor that affect consumer acceptance of insect-based foods.

Many strategies have been suggested to reduce food neophobia and increase acceptance of insect-based foods, among which processing seems to be the most promising (38, 83). Appropriate food processing and preparation techniques must be designed and implemented to obtain the benefits of insects. At all levels, including large-scale industrial, restaurants, cottage industries, and professional cuisine, as well as households, the processing is an important aspect of any meal or food item for the assimilation of insects into more standard cuisines. Processing preserves or improves the nutritional, organoleptic (texture, aroma, taste, etc.), and functionality of raw materials converted into food ingredients, while also destroying or removing potential safety hazards (93). Insect biomass processing is becoming increasingly necessary to meet the safe edible biomass standards while also developing effective techniques to reduce chemical and biological risks. Current insect biomass processing technologies rely on thermal (blanching, drying, boiling, freezing, chilling, and freeze-drying), mechanical (crushing, grinding, and milling), and fractionation processes (extraction, separation, purification, and centrifugation). These are well-developed and well-established in the food and feed industries (94). For example, the use of cold atmospheric pressure plasma processing in the postharvest chain for edible insects could aid in the creation of safe and high-quality insect-based products for the food and feed industries (95). Various food processing technologies such as oven, smoke, conventional air, freeze, microwave-assisted, and fluidized-bed drying methods, and ultrasound-assisted aqueous extraction, sonication, supercritical CO_2_ extraction, and dry fractionation can be used to improve the overall quality of insect-based products as well as helping in extracting nutritionally rich compounds from the insects for its application in developing new food products (96).

### Cell-cultured meat

To satisfy the growing demand for protein for an ever-increasing population, cultured meat is being considered a good substitute for meat. Cellular agriculture is an emerging field for the production of different products. Cultured meat, also known as clean meat or laboratory-grown meat, is a part of cellular agriculture and does not involve any livestock for the production of meat once the initial cells are obtained (13, 97, 98) although at some point new initial cells are needed.

**Figure 3:**
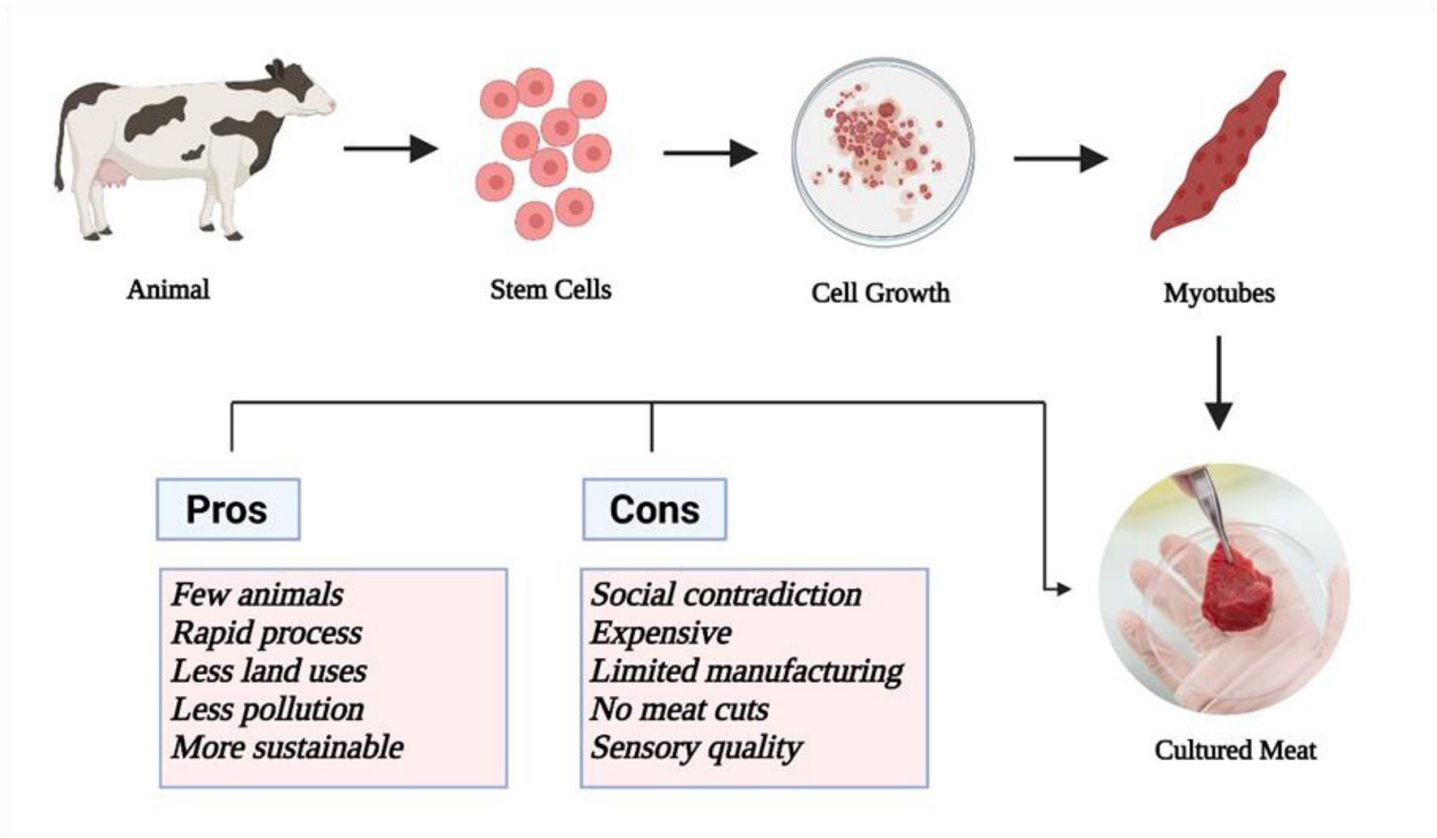
Process for the production of cellular meat with its pros and cons.

Cell-cultured meat is produced using tissue-engineering techniques. Different aspects of cultured meat give it an edge over traditional meat such as a lower use of environmental resources, higher nutritional value, lower risk of food-borne diseases, as well as avoiding issues associated with the slaughtering of animals (13, 99) In the cell-cultured meat process, a biopsy is taken from any living animal from which the stem cells are obtained. The stem cells can proliferate into different types of cells. These cells are cultured in a nutrient medium containing all the required growth factors, nutrients, and hormones. The cells, if directed to muscle growth, continue to grow and form myotubes with a length of about 0.3 mm. These myotubes are then placed in a ring that grows into a small piece of muscle tissue. A schematic diagram for their production is shown in Figure 3. These muscle tissues can further multiply to form more than a trillion strands. These muscle cells continue to grow in size and need to be attached to a scaffold that provides support and orientation (14, 100, 101).

The production process for cultured meat has various pros and cons. Cultured meat requires only a few animals to produce a large amount of meat through cell proliferation. The production process for laboratory-grown meat is rapid compared to natural processes. In addition, cell-cultured meat offers promising sustainability benefits. However, the cultured meat uses the blood of dead calves which is a controversial societal issue and negates the claims of being animal free and violates the religious traditions that do not permit the ingestion of blood or blood derivatives. The second major issue is that the use of this serum is expensive and increases the cost of production of laboratory grown meat. Currently only a limited range of meat cuts are available. Furthermore, the sensory quality of the meat is naturally affected by the type of animal including breed, growing conditions, feed, and many other factors, and to date has not fully imitated the flavor of the product it means to imitate. Additionally, laboratory-grown meat does not offer such diversification in terms of sensory quality. Therefore, there is a need to resolve the technical issues in the production of cultured meat. Additionally, there is a need to understand the safety aspects, optimization of cell culture methodology, and increase consumer acceptability. The acceptability of cultured meat by some religious authorities is still in question (14, 102, 103). A recent study showed that 35% of meat-eaters and 55% of vegetarians felt disgusted by cultured meat, as it is perceived as not being natural (104).

Despite the current limitations, the cell-cultured meat industry has recently been growing, especially in the past two years, with many companies being founded in North America, Asia, and Europe (105). Emerging innovations and Industry 4.0 technological advances (e.g., advances in biotechnology and 3D printing) are driving this trend, making it possible to accelerate the industrialization and commercialization process for cell-based products (4, 105). Among Industry 4.0 components, the role of 3D printing has been particularly highlighted, leading to many applications in different manufacturing fields, including cultured meat production (18, 106).

### 3D-printed foods

3D printing is being positively applied in different sectors of food production. The basic objective of 3D food production is to provide a highly structured food to the consumer. The main 3D food applications are based on the use of alternative ingredients, including different isolates from microorganisms, insects, food waste, and algae (107, 108). 3D printing can even be used to give a second life for plastic wastes (109), making it a promising approach for achieving sustainability and circular economy goals for food packaging. The major research on 3D food printing is being done in the USA, China, and Australia (108, 110, 111). The first use of 3D was for the creation of engineering prototypes, while the first 3D food was introduced commercially in 2015 (108, 112, 113). Presently, many technologies for 3D food printing are being used (39, 114, 115).

Different types of 3D-printed foods are available as shown in Table 1. The major 3D printed food-producing countries are China, the United Kingdom, Canada, Spain, the United States, and Poland (108). Three important categories of ingredients for food printing include native non-printable, printable, and alternative ingredients. The native printable materials (e.g., chocolate, icing, and butter) can be extruded from a syringe. In the case of non-printable traditional food materials (e.g., fruits vegetables, meat, and rice) different viscosity enhancers (e.g., starches, gums, and gelatin) are added after grinding for a smooth extrusion process. Proteins and fibers isolated from insects, agricultural waste, and algae are considered alternative ingredients and have different functional properties (108, 110, 113).

**Table 1:** Different types of 3D printed foods and consumer perception.

**Product**	**Consumer response**	**Reference**
Chocolates	The texture is too soft	(116)
	Higher levels of cocoa solids	
	Deliciousness	
	A very clever way of presenting tasty food	
Capsaicin candy	Nutritionally rich	(117, 118)
	Different shapes	
	Different flavors	
	Suitable for everyone	
	Helps in the prevention of numerous diseases	
Various types of meat products	Delicious	(18, 119)
	Different sizes	
	Rich protein source	
Pizzas	Highly delicious	(120)
	Healthiness	
Beef meat, hybrid meat analogs	3D printed meat	(18, 121, 122)
	Sustainable	
	Environmentally friendly	
	More durable	
	High cost	
	Potentially unappealing	

3D food printing technologies are producing demand-based foods that can address food-related diseases (e.g., diabetes and obesity) and personal nutritional habits (e.g., vegetarian and vegan). 3D food printing technologies can also have a role in the production of customized food products, eliminating undesirable substances, and making foods that are pleasant for the consumer. Other advantages include food waste reduction, innovation, and process digitalization (17, 107, 108, 110, 123).

3D printing can be considered one of the most important enablers of Industry 4.0. Recent advances and technological developments have accelerated innovation and strengthened the use of 3D printing for different applications. Improvements in simulation, modeling, software, and materials have led to the extension of 3D printing to 4D, 5D, and 6D printing (41). 4D food printing refers to the response of 3D-printed foods integrated with smart materials to external or internal environmental/human stimuli (e.g., temperature and pH), resulting in physical or chemical changes (e.g., color, flavor, or nutritional changes) in the products over time (17, 40, 41). An example of the application of 4D printing in food was recently given by Ghazal and others (124) who used red cabbage juice and vanillin powder for their 4D product to change color and flavor in response to an external or internal pH stimulus. Recently, more advanced and innovative printing technologies, including 5D and 6D printing have emerged, presenting new possibilities in food manufacturing (41). Compared to 3D printing that is based on three axes (X, Y, and Z) of movement, 5D printing allows products to be printed from five axes by adding two additional rotational axes (i.e., the rotation of extruder head and the rotation of print bed), enabling printing of complex shapes having curved surfaces. 6D printing combines 4D and 5D printing techniques, making it possible to print complex structures using smart materials (41).

However, there are many issues associated with the production of printed foods. Among different issues, the unusual appearance of 3D food is a significant concern. The acceptability of the 3D-printed foods is another important challenge that should be addressed (108). Several survey-based studies were not always encouraging (120). In addition, the safety aspects of 3D-printed food must be addressed. Production of 3D foods includes cooling and heating which make the food more susceptible to microbial growth. The sanitization process for the printer is important to reduce the microbial load in the final product (110, 114).

The market for 3D food printers is expanding for the production of various types of foods. This technology has various advantages in terms of health, economic, and environment aspects, with a potential to revolutionize food manufacturing. 3D printing technology could be a way to alleviate hunger through a more efficient use of the available foods and the use of alternative food sources. Further improvement in functional and nutritional properties of printed foods is expected with the advent of Industry 4.0 innovations, enhancing consumer acceptance. However, before large scale commercialization, the consumer confidence and safety aspects of 3D-printed food must be addressed.

### Use of by-products, ugly produce, and other sources (e.g., seaweeds and jellyfish) as food

Extensive research has been done to investigate new approaches to valorize food wastes and by-products and to explore new sources of food. Due to the growth of population and economic advances, larger amounts of agricultural and food wastes are produced at different stages of food production and consumption, causing different environmental problems (125, 126). Food wastes resulting from different food groups along the food supply chain were assessed and results showed that cereals, fruit, and vegetables were the food groups responsible for the highest amount of food wastes that occurred especially at the consumption stage (127).

Food waste valorization has garnered global attention as an effective approach in line with circular economy principles. Different strategies have been developed to reduce the waste resulting from the food industry and transform these wastes into resources (125, 128, 129). Food wastes and by-products can be rich in bioactive compounds and present important economic and environmental benefits. Different functional compounds may be extracted from food wastes and redirected to the food industry as ingredients or value-added compounds, to enrich products (47, 130). Proteins and amino acids, carotenoids and tocopherols, fatty acids, starches, oligosaccharides, soluble fibers, flavonoids, aromatic compounds, and different vitamins are examples of functional compounds extracted from different by-products and “ugly” produce and used to enrich different foods (131–134).

However, most of the current waste valorization strategies are developed only at laboratory scale (127). Additionally, consumer acceptance remains one of the main barriers that prevent commercialization of products and compounds extracted from food wastes and ugly produce. Research shows that abnormal appearance and nearing expiration date of food products can reduce consumer willingness to accept these products (48). In a recent study, the main drivers of intention to purchase products with a by-product, namely grape pomace powder, were evaluated (135). The results indicated that informing consumers positively of the presence of this by-product in food formulation enhanced the consumer acceptance of the product.

Jellyfish and seaweed have been highlighted in many studies as potential future foods (136–145). Jellyfish are marine invertebrates that are capable of growing in various environments (such as cold and warm waters, along coastlines, and in deeper waters) to form large blooms (146, 147). Interestingly, many reports indicated that the availability of jellyfish seems to increase with climate change, such as global warming (137, 147). These sustainable marine bioresources are valued for their reported health benefits showing high potential for use in food, feed, pharmaceutical, and other biotechnological applications, promoting their cultivation (146). Seaweeds are plant-like organisms that belong to brown (Phaeophyta), red (Rhodophyta), and green (Chlorophyta) algae (147, 148). These renewable sources of food have gained increased research and consumer interest in recent years due to their nutritional properties (e.g., high content of proteins, vitamins, minerals, and bioactive compounds) and their sustainability characteristics (e.g., fast growing with no fertilizer or pesticides), making them significant contributors to global food security (141, 142, 147).

Valorization of food wastes and by-products and exploitation of novel food sources take advantage of recent technological advances and the rapid spread of the concept of Industry 4.0. IoT, digital technologies, such as AI and digital twins, and other Industry 4.0 components are being applied to reduce or valorize food wastes and by-products, providing important environmental and economic benefits (62, 149–152). For example, emerging innovations in the field of algae biotechnology enable the development of low-cost production with exciting opportunities of automation through the application of IoT and other technological advances (145).

Developments in nanotechnology have provided many promising applications in the food industry, such as the use of food wastes and by-products in different sustainable food packaging strategies (153). Nanotechnology was used to reduce wine waste in obtaining new food ingredients and sustainable packaging with improved stability and bioavailability of the phenolic compounds (154). Grape pomace and broken wheat were used as printing material to produce functional cookies with enhanced nutritional value and antioxidant properties (155). The results showed that this sustainable approach led to food products with customized shapes and a higher content of proteins and dietary fiber.

Most of the extraction methods that are available industrially possessed several bottlenecks, such as using strong acidic solutions and high temperatures, including boiling water, leading to negative impacts on the sensory and nutritional quality of the extracted compounds and decreased consumer acceptance. Moreover, these extraction methods depend on different factors such as solvent properties, reaction temperature, pH, time of reaction, and the ratio between solvent and solid material (156–158). One the other hand, emerging green technologies, such as supercritical fluids, cold plasma, pulsed electric field, ultrasound, and high pressure processing have been studied and suggested as alternatives to conventional extraction methods. These techniques have a high potential to improve or maintain sensory and nutritional properties of foods, thus increasing their positive perception by consumers (25, 125, 158).

### Personalized diet

A person's state of health can be improved through an individualized or personalized dietary approach. Healthy dietary choices may help to substantially reduce the occurrence of obesity and non-communicable diet-related diseases such as cancer, type-2 diabetes mellitus, metabolic syndrome, and cardiovascular diseases (159). Existing policy emphasizes prevention through personalized health promoting interventions, which have been shown to be successful in changing healthy behavior of consumers (160, 161). Digital technologies sustain individualized health promoting interventions by providing a personalized approach to health supporting activities that are easily accessible and cost effective (161). With the current breakthroughs in the decoding of the human genome and various applications of genomics, epigenomics, proteomics and metabolomics in medicine and nutrition, the modern personalized diet goes far beyond previous customized nutritional advice based on diet, age, sex, body mass index (BMI), physical activity and the clinical picture (160, 162). An individual's response to any food and/or food component is assigned to a number of factors including overall state of the health, the genetic profile and physiological environment (163). To minimize side-effects arising from the consumption of physiologically unsuitable food products by an individual, identification of the factors that may predispose the individual to specific diseases as the result of the diet leads to a proposed customized diet. Knowing the sequence of the human genome, it is feasible to develop a personalized diet regime that can be used by each individual based on his/her genetic make-up.

Personalized diet is important for the development of foods that may be used as a “drug” for the prevention of a specific disease affecting that individual. The use of personalized diet will make dietary interventions more efficient by simply changing the diet that have been proven ineffective in certain genotypes (16, 164). The first documented attempt to develop personalized nutrition practices was the ancient medicine system of Ayurveda, that goes back to 1500 B.C.E. Ayurveda as a traditional medicine system, represents a set of comprehensive healthcare practices involving medicine, nutrition, science, and philosophy (165). According to Ayurveda, predisposition to a disease depends on an individual's basic constitution (Prakriti) which requires a certain diet and health practices to avoid (166). With help from modern predictive medicine, Ayurveda's efforts have been directed toward personalized nutrition based on prospective disease and markers for their conditions. Individuals from the three basic constitution types as defined by Prakriti type do show major differences for each type at the genome-wide expression level, as well as their biochemical and hematological parameters including lipid profiles, liver function tests, and hemoglobin content (165). Since the genetic expressions are strongly affected and may be altered by diet, an unhealthy lifestyle and environmental factors, the dietetic principles of Ayurveda have been claimed to help maintain genetic expression (167, 168).

A modern relook into the basics of Ayurveda dietetics had a strong relation to epigenomics, proteomics and metabolomics, which led to the emergence of the concept of personalized nutrition called Ayurnutrigenomics (168). This emerging field of research may show the possibilities of smart dietary choices that will help to prevent non-communicable diseases and lifestyle disorders caused by gene alteration through a fresh insight into specific dietary recommendations based on the genotype of individuals (167–169).

The ability of food components to interfere with molecular mechanisms on a genetic lever has raised consumer interest in considering personalized diet. The consumers are becoming more intent on matching their own genotypes and phenotypes to a diet that will help achieving desired physiological outcomes (16). Personalized diet can, therefore, be viewed as a solution to consumers' needs for health promoting diets and dietary advice (170). However, personalized nutrition needs the development of personalized food products. Working with the variations in individual needs based on biological characteristics of the body, personalized nutrition provides recommendations on types of foods and their optimal intakes. Personalization of food products requires knowledge of their nutrient composition and greater understanding of all possible interactions and impact of micro- and macronutrients on the individual's health (43).

To make personalized diet efficient for an individual, relevant personalized food products must be developed and made available. Current production of personalized products includes not only knowing the genomics of consumers, but also characteristics of nutrients in a food matrix, development of food products with specific functional properties (43), and application of advanced technologies that incorporate elements of Industry 4.0 (30). Therefore, mass production of personalized food products is currently not feasible without wider applications of Industry 4.0 technologies, since it is a comprehensive, laborious, and time-consuming process requiring specific knowledge in the fields of medicine, genomics, nutrition, and food technology. The involvement of Industry 4.0 is required for wider use of the information technologies needed to model and describe the biological processes in medicine and nutrition, including statistics and data processing, genomics, epigenomics, proteomics and metabolomics, which would help to accelerate the development and spread of personalized foods, as well as increase consumer awareness and acceptance of personalized foods. Therefore, personalization will also require consumer input to define food preferences within potential choices (43). Several attempts have been taken to develop personalized foods that could match the genetics of an individual with direct involvement of that consumer. During these trials, European consumers, particularly with health issues, showed an openness and interest in the food personalization research (171). There were also attempts to develop personalized foods by selecting and combining food ingredients in accordance with personal requirements and preferences, thus enabling consumers to contribute to the personalization of food while feeling satisfied and socially involved in the process (172, 173).

Thus, personalization of food is a complex process that requires the development of personalized food products based on the nutritional profile of raw material and genomics of a specific individual, taking into account all the interactions between nutrients and other compounds in the food matrix during processing and storage, and their influence on health and consumer acceptance of the final products.

### Fortification and gene editing of foods

Food fortification is a widely used strategy to address the problem of nutrient deficiency and prevent malnutrition. As traditional fortification approaches, i.e., the direct addition of nutrients (e.g., vitamins and minerals) to foods during processing, can be challenged by bioavailability issues, the development biofortification has been accelerated in recent years. The development of fortification techniques, microencapsulation and stabilization technologies, and the intervention of genetic engineering and breeding pave the way to develop various foods (especially staple food crops, such as rice and dairy products, such as cheese) with a higher nutritional value and/or a greater content of health-promoting compounds compared to traditionally fortified foods (174–176).

**Figure 4:**
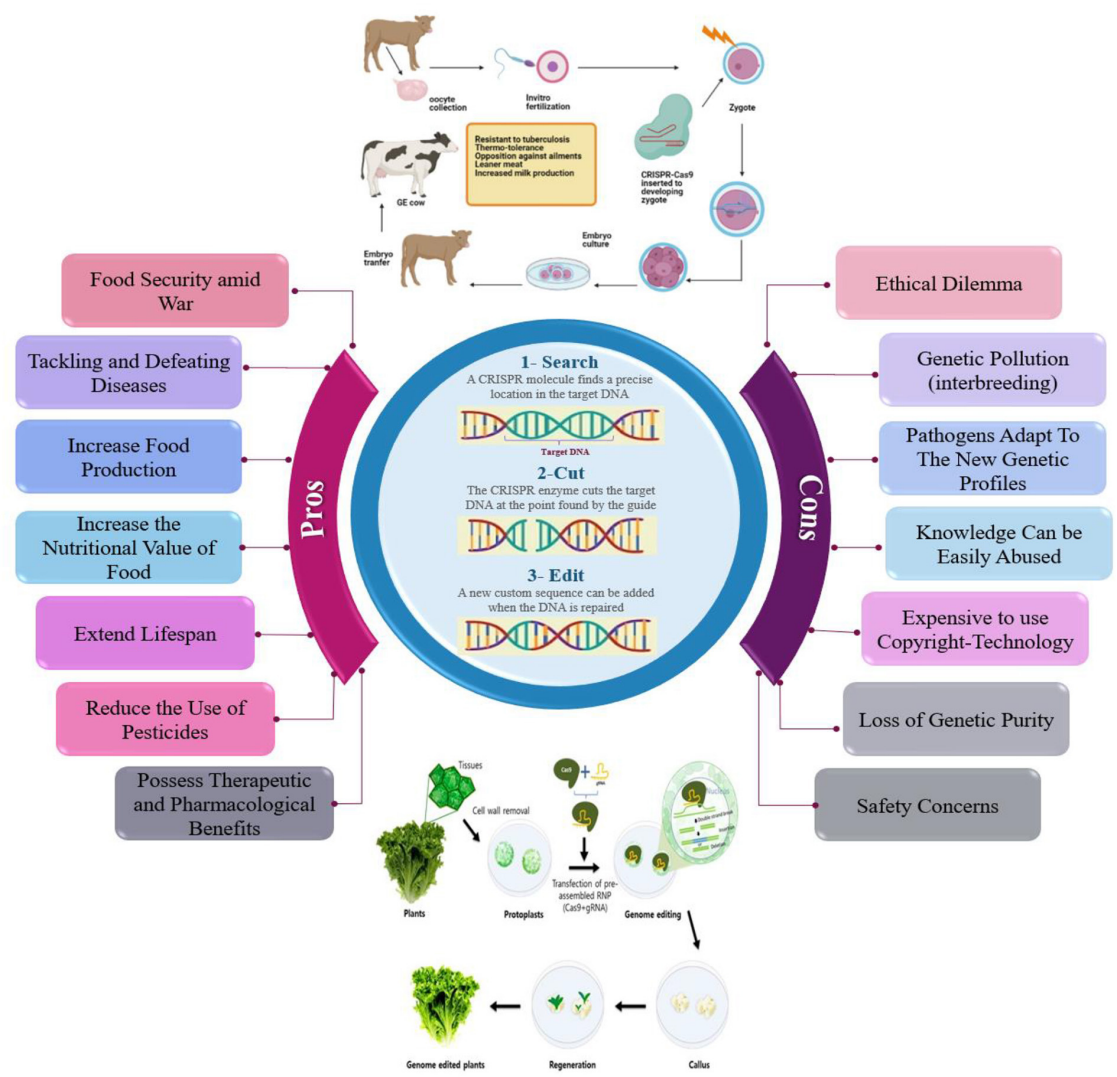
Process for the gene-editing with its pros and cons.

Genome editing has been applied as an alternative approach to improve the nutrient contents of crops and livestock products. Figure 4 shows the process for the production of cellular meat with its pros and cons. Scientists starting in 2003 have developed gene-editing (GE), which allowed them to develop modified crops and livestock with high performance across a variety of features including both abiotic and biotic stresses (20). GE is the capacity to make exact alterations to a live organism's DNA sequence, thereby modifying its genetic composition. This method works by using enzymes, notably nucleases, designed to target a specific DNA sequence. These enzymes function like scissors, cutting the DNA at a specific spot, and allowing the removal of existing DNA and the insertion of replacement DNA (21). Among the effector nucleases used for GE are meganucleases (MegN), transcription activator-like effector nucleases (TALEN), and zinc-finger nucleases (ZFN). Discovered in 2012 (21), clustered regularly interspaced short palindromic repeats (CRISPR-Cas9) allowed molecular scissors to precisely target a gene in the genome. The procedure first requires identifying a gene responsible for a particular function that requires editing. To edit DNA, a guide RNA (ribonucleic acid) is constructed and the Cas9 enzyme cuts the specified sequence at a designated spot. After the cut, certain functionalities may be added or modified, and the cell can be restored using enzymes.

In an organism, the alterations are accomplished once the guide RNA and Cas9 enzyme are eliminated (177). Overall, GE breeders achieve a particular genotype that occurs naturally at a low frequency, and Cas9 is a crucial differentiator for both breeders looking to establish new lines of animals and regulators who understand that the results are similar to natural mutations. The use of GE methods has opened up many possibilities for generating crops and animals that can better deal with the issues of food and nutrition security. A few examples:

Rice has been a staple for more than half the world's population. Therefore, the first study using CRISPR-Cas9 technology focused on rice GE (178). Drought and salt are two critical abiotic factors that influence rice that GE might address. The use of CRISPR-Cas9 to knockout OsRR22, a gene linked to salt sensitivity in rice increased its success with high saline conditions (0.75% NaCl) without reducing grain production, plant biomass, or grain quality. These GE lines were 19% shorter, whereas wild-type plants were 32% shorter with salt. Likewise, GE plants had no significant changes from the unedited plants in the absence of saline and had considerably less severe biomass decreases due to salt exposure. Saline tests were done in greenhouses, and overall agronomic performance was assessed in the field. Compared to wild-type plants, GE plants had substantially less severe biomass losses due to salt exposure (179). Other rice editing efforts have resulted in early maturing rice that is more suitable for cultivation in the northern hemisphere, where it needs a longer growing period and colder temperatures (180). Rice plants were GE using CRISPR-Cas9 to target the flowering-related genes Hd2,4, and 5, resulting in plants that bloomed considerably quicker than their wild-type counterparts.

In the future, when temperatures and other climatic conditions in tropical areas make agriculture less productive, early flowering plants may be better suited. Aside from being adapted to water shortages, the early blooming may also reduce the amount of cumulative water needed to grow to harvest. GE technologies may assist with knock-ins as well as knockouts. To improve drought tolerance, researchers used CRISPR-Cas9 to place a promoter into a particular maize gene. An alternative maize promoter was placed before ARGOS8, a drought-tolerant gene. This precise insertion resulted in higher grain yields with water stress during flowering while preserving normal growth conditions. This method is an intragenic strategy using GE in which a native maize genetic sequence was placed at a new locus to improve plant tolerance to the abiotic stressor (181).

The development of disease resistance in pigs has also benefited from GE. Two genes, CD163 and CD1D were knocked out (182). The former is essential for PRRS viral infection, whereas the latter is involved in innate immunity. The Cd163 knockout pigs were tested for PRRS resistance and had no symptoms when infected. On the other hand, the wild-type offspring had severe symptoms and had to be euthanized (182). By using CRISPR-Cas9 GE to knock out CD163, pigs might become immune to PRRSV (183). CRISPR-Cas9 has been used to knock-in resistance to the classical swine fever virus (CSFV) at the Rosa26 gene. This locus is a suitable target for transgenic insertion because of its widespread and high expression, and the absence of any gene-silencing effects (184). The gene-edited pigs were CSFV-resistant, but all wild-type pigs died each time. The *C. elegans* fat-1 gene was inserted into the Rosa26 locus in pigs, providing a proof-of-concept to illustrate the feasibility of concurrently boosting the nutritional value of pork while raising general disease resistance since fat-1 is involved in both disease resistance and the nutritive quality of meat (184). Also, the ANPEP (aminopeptidase N) gene was knocked out using CRISPR-Cas9 GE to impart resistance to coronavirus infections (185).

Consumers' acceptance or rejection of food produced from GE crops may have significant economic consequences at all stages of the food system. Global agreement on the safety and regulation of GE crops is non-existent, and this is a serious and significant problem. GE in food production is accepted by many nations and areas, while Europe and New Zealand have adopted a more cautious approach (186). In a study of around 10,000 individuals done by the University of Tokyo, 40 to 50% of respondents said they did not want to eat GE crops or animal products (186). Another study investigated people's attitudes toward GE in food plants and animals. People tended to show more positive attitudes toward GE plants than GE animals. Their acceptance was stronger for biotechnology medical applications than agri-food applications (44). Consumers have historically reacted negatively to genetically modified products (GMO) because of their perceived “unnaturalness,” and GE foods may encounter the same problems. Food that is more nutrient-dense, environmentally and animal welfare-friendly and cost-effective may be created by GE and overtime become consumer acceptable.

GE discussions need to be framed so as to enable the public to participate in the discussion, manage any misunderstandings, and maintain consumer trust. GE products may also need labeling that is clear and accurate.

### Hydroponics, aquaponics, and other indoor vertical food production systems

Agriculture-based food production growth is now much lower than the rate of population expansion, which is a concern. As a result, more agricultural production systems must be implemented to improve and achieve expected future food supply needs. Alternative forms of farming systems have become more popular. Hydroponics, aquaponics, and other indoor vertical farming systems are some of the primary sectors where global agricultural output may be improved as growing conditions can be better managed. Table 2 shows the pros and cons of hydroponics, aquaponics, and aeroponics systems. Hydroponics is a type of horticulture and a subset of hydroculture that includes mineral fertilizer solutions in an aqueous solvent to grow plants, mainly crops, without soil (187). Any crop may be grown hydroponically, but the most frequent are leaf lettuce, celery, cucumbers, peppers, tomatoes, strawberries, watercress, and various herbs (24). Aquaponics is an indoor vertical farming system combining aquaculture (fish farming) and hydroponics. In aquaponics, farmed fish waste provides nutrients for hydroponically grown plants, which in turn clean the water for the fish. This ensures a closed-loop, long-term feeding supply. Because few pesticides and herbicides are non-toxic to fish, aquaponics production relies on organic pest and weed management (22, 188, 189). Aeroponics is a soilless revolutionary farming system that allows growing plants in the air, where plants' roots are suspended in a mist of nutrient solution (49, 190). This system is well suitable to automation, digitalization, and other advanced technologies. For example, an automated IoT-based aeroponics system, with remote data monitoring, including sensors measuring temperature, humidity, pH value of the water, and the light exposure, has been developed (23).

**Table 2:** New food production systems.

**Type of agriculture technique**	**Definition**	**Advantages**	**Disadvantages**
Hydroponic	Cultivation of plants without soil using mineral nutrient solutions in aqueous solvents	No soil involved and no need for soil preparing or testing Optimal use of location (20% less space for growing) Complete control over climate Conserving water by reusing it (20% less water than traditional agriculture) Optimal use of nutrients Zero weeding, mulching, etc. Faster growth rate Total control over the nutritional balance Fewer pests and ailments since the environment is sterile	Constant monitoring is required Water-based microorganisms may readily infiltrate the system All plants in the system will be impacted if a disease emerges If the system fails without soil to act as a buffer, plant death will ensue quickly Risks of water and electricity failure Requires a high level of technical knowledge Some plants are difficult to grow hydroponically (such as potatoes) Could be expensive
Aquaponics	A food production system integrating aquaculture and hydroponics to grow fish and plants together in one system	Efficient use of water and nutrients Organic fertilization Environmentally friendly Produce the highest yield on a field Smart vertical farming Consistent with circular agriculture	Limited crops High initial cost High consumption of electricity Unsustainable fish food The system must be professionally installed Unexpected failure
Aeroponics	A method of growing plants without any growing medium where the roots are suspended in the air, and nutrient solutions are delivered to the plant using a fine mist or spray	Completely controlled environment Uses fewer resources, e.g., 90% less water than traditional farming Saves considerable space and soil Fast growth and high yield As roots grow in the air, there is no physical medium inhibiting a plant's expansion Growth environment can be pest- and disease-free	Requires advanced machinery and equipment to operate Expensive initial investment Dependency on electricity Requires more monitoring and maintenance compared to the other systems Nutrient content of the solution must be monitored carefully and constantly Extremely sensitive system

Integration of Industry 4.0 technologies to food production systems is termed smart farming or precision farming (190–192). Application of Industry 4.0 innovations (e.g., robotics, IoT, drones, satellite imagery, and smart sensors) in food production systems can improve productivity and enable data collection and aggregation, providing further improvements of precision technology and possible solutions to various problems, which could not be solved with traditional farming systems (24, 191, 192). A few examples:

The application of drones is now being investigated across various production sectors, including agricultural supply chains, providing relevant opportunities to overcome challenges (193). The application of Industry 4.0 technologies to aquaponics is termed Aquaponics 4.0, referring to a digital aquaponics farm that involves remote monitoring and control of ecosystem parameters, a high degree of automation, and intelligent decision-making to ensure high crop yields and quality (22, 188).

Despite the global spread of these alternative farming systems, only a limited number of studies discuss the overall health of the plants grown using hydroponics, aquaponics, and aeroponics, and the consumer acceptance of these foods. A study of hydroponics and aquaponics cultivation showed that >60% of consumers are generally unfamiliar with these systems and their products (194, 195). Overall, three major categories of features allow classifying consumers' attitudes and beliefs, and identifying prospective buyers of these farming products. These factors include (i) the consumers' personal and sociodemographic characteristics and their prior knowledge; (ii) the consumers' willingness to pay some percentage for locally produced or pesticide/herbicide and antibiotic-free products before the concept of these cultivations was introduced; and (iii) the consumers' willingness to pay some percentage for products after the concept of these systems was introduced and it meets consumers' main values regarding those products.

## Conclusions

The potential of the Industry 4.0 revolution technologies to enhance eight food trends (namely, plant-based, insect-based, cell-based, 3D-printed, personalized, and GE foods, as well as foods resulting from by-products and ugly produce, and new production systems) were explored in this narrative review. According to the Scopus database, there has been a significant increase in the number of publications and citation on these food trends. The role of emerging technologies in promoting more acceptability and consumption of proteins from these traditional (such as plant- and insect-based foods) and innovative (such as GE foods, cell-based meat, and 3D printed products) alternative sources has been highlighted. The main outcome of this review paper is to broaden readers understanding of the applicability of emerging and innovative techniques to achieve a shift to digital and ecological transitions toward greener, healthier, and more sustainable diets.

The first, second, and third industrial revolutions were characterized by mechanization, electrification, and information advances, respectively, while automation, digitalization, hyperconnectivity, as well as fusion between physical, digital, and biological worlds are the main features of the ongoing fourth industrial revolution, called Industry 4.0. Industry 4.0 is a huge umbrella term that includes artificial intelligence, big data, smart sensors, robotics, and block chain, to name a few.

Traditional food production systems, such as livestock farming, have been identified as a contributor to climate change and unsustainable development. There is therefore a search for alternative proteins that have comparable health and sensory characteristics to traditional animal-based products but with a lower environmental footprint. PB sources have traditionally been the most investigated, especially PB proteins from oilseeds (e.g., rapeseed and hemp), legumes (e.g., lentils, beans, and peas), and cereals (e.g., wheat and rice), as well as fruits and vegetables for use as food and feed. Although in use since antiquity, an increased consumer interest in these foods has recently emerged with a growing number of vegans, vegetarians, and flexitarians. In addition, a variety of PB meat, fish, milk, and egg analogs have been seen as a promising sustainable approach to reducing the consumption of meat and other animal-based proteins (10, 34, 35). A challenge to consumption of PB foods is many of their poor nutritional and functional properties. However, this issue can be solved by blending different types of proteins from various sources and optimizing processing conditions, thus improving protein quality, digestibility, and bioavailability (32, 33). Additionally, current research shows that Industry 4.0 innovations and emerging processing technologies could help to improve their nutritional and technological functionality as well as their sensory perception. The recent innovations and advancements could be a major driver in convincing consumers to rely more on PB diets.

The popularity of insect-based diets has been increasing spurred by the increased awareness and demand of consumers for sustainable alternatives to animal proteins. Insects could be the food of the future, but currently most consumers do not seem willing to adopt the consumption of insect proteins. Therefore, continued efforts will be required to change the minds and behaviors of consumers. Industry 4.0 technologies and new innovations in processing technologies and analytical approaches such as metabolomics (36) could help to improve the sensory and techno-functional properties as well as digestibility of insect-based foods, enhancing their acceptability and making them more appealing to consumers. Many insect-based products (e.g., insect flour, insect protein powders, and insect protein hydrolysates) can be prepared from numerous edible insect species (especially crickets and mealworms) and can be used as snacks or ingredients to produce other food or feed. Insect foods are nutritious, cheap, and sustainable as less food, land, and water are required for insect breeding than raising cattle or other livestock. Although positive aspects of insect proteins regarding both nutritional and environmental issues has decreased entomophobia, intensive studies on safety, hygiene and toxicity, marketing strategies, and governmental regulations should be done to accelerate consumer acceptance of these alternative portion sources.

Cell-cultured meat and related products (e.g., seafood and poultry) seem to be one of the most promising and revolutionary strategies to achieve environmental sustainability and improve animal welfare, hence the large number of patents and publications (13). As the process of production occurs in the laboratory, water and land requirements as well as greenhouse gas emissions are low. Although the technique has the potential to disrupt and transform the whole agricultural and food industry, it is still costly and production at a large scale has not yet been implemented. Moreover, concerns about the naturalness, ethical issues, and safety of cultured meat and related products still exist among consumers, while studies on health benefits, funding resources and appropriate regulatory pathways are still required (14, 196).

3D printing has been accepted by many scientific and industrial areas, including the food industry. This technique could enable producing on-demand, complex, and customized (e.g., shapes, sizes, tastes, texture, and nutritional properties) food products to satisfy a range of consumer preferences. Several 3D printing techniques have been developed, with extrusion-based printing being the most common. The last few years have seen significant progress in this field, with the emergence of new smart materials, new technologies, and significant innovations, accelerating the move toward more advanced and innovative additive manufacturing technologies, including 4D, 5D, and 6D printing (40, 41). Although the scope of current application is limited to the decoration and fabrication of a few food products such as chocolates, cookies, and cakes (39), further improvement in functional and nutritional properties of printed foods is expected with the advent of Industry 4.0 innovations, enhancing consumer acceptance. Owning a personal food printer at home is probably likely in the not so distant future. One of the possible applications of food printing is personalized foods (or personalized nutrition) as food can be specifically printed to meet an individual's health-nutritional needs, including medicinal and nourishment requirements (16, 17).

Industry 4.0 technologies should be considered to reduce the environmental impact of food production systems and achieve zero-waste. According to FAO, a huge amount of food by-products and ugly produce are wasted or lost every day. Increased concerns about environmental sustainability are driving the growing interest in better uses of food waste and by-products, and ugly produce. Technological innovations and scientific advances along with education could help consumers accept the hidden beauty of ugly food, thus reducing food waste and contributing to food sustainability.

Gene editing methods have opened up many possibilities for generating crops and animals with desired traits. New gene editing tools (e.g., CRISPR-Cas9-based GE) are being rapidly developed, taking advantages of recent progress in genetic engineering. GE is efficient and can enhance product quality and increase resistance against diseases and pests with a low risk of off-target effects. GE could bring about new possibilities in agriculture and biomedicine but consumer acceptance of GE products is still low. High-productivity food systems, including hydroponics, aquaponics, and other indoor vertical farming methods, are occurring in smart controlled food production environments. These smart or precision farming methods are becoming better understood to fulfill future food demands.

## Future perspectives

While this review is not an exhaustive overview of all emerging food trends, eight of the more pertinent ones, from food technological advances perspectives, were discussed. Each of these emerging food trends has been fostered by the greater use of Industry 4.0 technologies and recent advances in many fields of food science and technology. Innovative solutions based on Industry 4.0 enablers (such as AI, smart sensors, and robotics) can be used to increase agriculture productivity, optimize production conditions, and reduce waste and loss, accelerating the green and digital transition of future food production systems. The interest in traditional animal-proteins alternatives, including plant-based foods and insects and more recent food trends, such as cell-cultured meat, 3D-printed, fortified, and gene-edited foods are likely to continue growing in popularity in response to the increasing consumers' awareness regarding the environmental impact of food choices. With the ongoing rapid technological advances in physical, biological, and digital worlds, other food trends are expected to emerge in the future.

Although the concept of Industry 4.0 may have previously had greater significance to other industries, the opportunities for the agriculture and food industry sectors are also enormous. Wider applications of Industry 4.0 technologies in the agriculture and food industry could enable the production of foods with higher quality and affordability, and lower environmental impact. Innovative technologies provide opportunities for improving sensory and nutritional properties of foods, thus increasing their positive perception by consumers, which in turn could enhance food sustainability and contribute to addressing the issue of global food insecurity.

## Author contributions

Conceptualization, methodology, and writing—original draft preparation: AH. Writing—original draft preparation: JC, MT, AVR, OB, GN, YJ, FS, MA, PM, and AK. Writing—review and editing: AH and JR. Funding acquisition: JC. All authors contributed to the article and approved the submitted version.

## Funding

The research was funded by the Norwegian University of Science and Technology, Department of Biotechnology, and Food Science.

## Conflict of interest

Author MT was employed by Centre for Innovative Process Engineering (CENTIV) GmbH.

The remaining authors declare that the research was conducted in the absence of any commercial or financial relationships that could be construed as a potential conflict of interest.

## Publisher's note

All claims expressed in this article are solely those of the authors and do not necessarily represent those of their affiliated organizations, or those of the publisher, the editors and the reviewers. Any product that may be evaluated in this article, or claim that may be made by its manufacturer, is not guaranteed or endorsed by the publisher.
